# Epilepsy, cognitive deficits and neuroanatomy in males with *ZDHHC9* mutations

**DOI:** 10.1002/acn3.196

**Published:** 2015-04-09

**Authors:** Kate Baker, Duncan E Astle, Gaia Scerif, Jessica Barnes, Jennie Smith, Georgina Moffat, Jonathan Gillard, Torsten Baldeweg, F Lucy Raymond

**Affiliations:** 1 Department of Medical Genetics, Cambridge Institute for Medical Research, University of CambridgeCambridge, United Kingdom; 2 MRC Cognitive and Brain Sciences UnitCambridge, United Kingdom; 3 Department of Experimental Psychology, University of OxfordOxford, United Kingdom; 4 Speech and Language Therapy Team, Cleft.Net.East, Addenbrookes HospitalCambridge, United Kingdom; 5 Department of Radiology, University of CambridgeCambridge, United Kingdom; 6 Developmental Neuroscience Programme, Institute of Child Health, University College LondonLondon, United Kingdom

## Abstract

**Objective:**

Systematic investigation of individuals with intellectual disability after genetic diagnosis can illuminate specific phenotypes and mechanisms relevant to common neurodevelopmental disorders. We report the neurological, cognitive and neuroanatomical characteristics of nine males from three families with loss-of-function mutations in *ZDHHC9* (OMIM #300799).

**Methods:**

All known cases of X-linked intellectual disability (XLID) due to *ZDHHC9* mutation in the United Kingdom were invited to participate in a study of neurocognitive and neuroimaging phenotypes.

**Results:**

Seven out of nine males with *ZDHHC9* mutations had been diagnosed with epilepsy, exceeding epilepsy risk in XLID comparison subjects (*P* = 0.01). Seizure histories and EEG features amongst *ZDHHC9* mutation cases shared characteristics with rolandic epilepsy (RE). Specific cognitive deficits differentiated males with *ZDHHC9* mutations from XLID comparison subjects and converged with reported linguistic and nonlinguistic deficits in idiopathic RE: impaired oromotor control, reduced verbal fluency, and impaired inhibitory control on visual attention tasks. Consistent neuroanatomical abnormalities included thalamic and striatal volume reductions and hypoplasia of the corpus callosum.

**Interpretation:**

Mutations in *ZDHHC9* are associated with susceptibility to focal seizures and specific cognitive impairments intersecting with the RE spectrum. Neurocognitive deficits are accompanied by consistent abnormalities of subcortical structures and inter-hemispheric connectivity. The biochemical, cellular and network-level mechanisms responsible for the *ZDHHC9*-associated neurocognitive phenotype may be relevant to cognitive outcomes in RE.

## Introduction

Genetic diagnosis is now possible for an increasing proportion of individuals with neurodevelopmental disorders, including intellectual disability and childhood epilepsies. Postgenomic investigation of rare, recurrent Mendelian disorders has the potential to identify specific phenotypic correlates and highlight novel neurodevelopmental mechanisms relevant to common, complex conditions. Considerable progress has been made in identifying genes responsible for X-linked intellectual disability (XLID), and more than one hundred recurrent causes have been reported.^1^,^2^ Inherited mutations in *ZDHHC9* were identified in four of two hundred and fifty families with XLID via a systematic screen of coding exons and splice junctions of all X chromosome genes.^3^ *ZDHHC9* encodes a palmitoylation enzyme involved in reversible lipid modification and cyclical intracellular localization of neuronal and non-neuronal substrates.^4^ Recent functional analysis confirms that all XLID-associated mutations reduce ZDHHC9 enzymatic activity^5^ however, the cellular and developmental mechanisms leading from reduced palmitoylation to XLID are not understood.

In common with many gene discovery studies, detailed neurological histories, cognitive evaluations and neuroimaging were not available at the time of the initial *ZDHHC9* mutations report. To address this limitation, we carried out a neurocognitive phenotyping study of all UK-based cases diagnosed with mutations in *ZDHHC9,* the results of which are reported in the current paper. The first phase of this study uncovered evidence that *ZDHHC9* mutations are associated with susceptibility to focal seizures sharing features with rolandic epilepsy (RE; also known as Benign Childhood Epilepsy with CentroTemporal Spikes, BECTS), the most frequently diagnosed epilepsy syndrome of childhood.^6^,^7^ Having identified this unexpected association, we posed two targeted hypotheses. First, we predicted that males with *ZDHHC9* mutations might share specific cognitive characteristics with RE^8^,^9^ and that these specific deficits would discriminate *ZDHHC9* mutation cases from IQ-matched comparison subjects with mutations in other XLID genes. We assessed speech and language functions known to be impaired in a significant proportion of individuals with RE and to persist after seizure remission.^10^–^12^ We also assessed aspects of nonlinguistic attention, because impairments in attention have been consistently reported in RE and may reflect altered network maturation relevant to long-term cognitive outcomes.^13^,^14^ Second, we predicted that *ZDHHC9* mutations might be associated with consistent structural brain abnormalities, in contrast to heterogeneous findings in the idiopathic (mixed etiology) RE population.^15^

## Methods

### Study population

Ethical approval was granted by the Cambridge Central Research Ethics Committee (11/0330/EE). Eligible participants were males >6 years in whom a pathogenic variant in an XLID gene had been identified (http://goldstudy.cimr.cam.ac.uk/). The *ZDHHC9* case group comprised nine males from GOLD families 31, 152 and 576,^3^ age range 8.6–41.3 years (mean 27, SD 14). Neurological and behavioral characteristics were compared to 26 males with XLID and pathogenic mutations in other published genes (XLID control group), age range 9.6–54 years (mean 28, SD 13): *AP1S2* (*n* = 3), *CUL4B* (*n* = 5), *DLG3* (*n* = 3), *HUWE1* (*n* = 3), *PAK3* (*n* = 5), *PTCHD1* (*n* = 1), *SYP* (*n* = 2), *UBE2A* (*n* = 1), *SLC9A6* (*n* = 2), *OPHN1* (*n* = 1). Cognitive testing was carried out for *ZDHHC9* cases (*n* = 8) and XLID controls (*n* = 9) matched in age (Mann–Whitney *U P* = 0.236) and severity of ID (Vineland Adaptive Behaviour composite score, Mann–Whitney *U P* = 0.195). Research MRI was carried out for *ZDHHC9* cases (*n* = 7) and individually age-matched (±2 years) male controls with no history of neurological illness or cognitive impairment (*n* = 7).

### Neurological and behavioral assessments

Structured medical interview was supplemented by review of medical records and clinical investigations including EEG where available. Epilepsy phenotypes were classified according to ILAE guidance.^16^ Neurological examination was carried out by a qualified pediatrician and video-taped for later review. Behavioral questionnaires were completed by a close family member or professional carer. Global ability and adaptive functions were assessed via Vineland Adaptive Behaviour scales,^17^ everyday language skills via age-appropriate Communication Checklists,^18^,^19^ and behavioral problems via age-appropriate Developmental Behavior Checklists.^20^,^21^

### Cognitive testing

Assessment tools were selected to be comparable to previous investigations of idiopathic RE, and appropriate for children and adults with ID. The protocol tested general intellectual abilities (WASI-II^22^), auditory processing (Filtered Words, Competing Words from SCAN-A^23^), receptive language (Word Classes, Concepts and Directions from CELF-IIIR^24^) and expressive language (Rapid Automatic Naming, Formulating Sentences from CELF-IIIR^19^). Speech and nonspeech oromotor functions were assessed via the Verbal Motor Production Assessment for Children (VMPAC).^25^ VMPAC assessments were video-rated by two speech and language pathologists blind to genetic diagnosis.

Three nonlinguistic visual attention tasks (detection, oddball, Go/NoGo) analogous to continuous performance tests were administered according to a method previously described.^26^ In each task, participants are instructed to respond via a button box as quickly as possible to the same easy-to-discriminate visual targets (high-contrast Gabor patches) presented on a laptop computer. Each task begins with slow practice trials followed by real-time practice and 1 min test blocks with fifteen targets per block. Interstimulus interval was fixed at 300 msec, stimulus duration 50 msec. Auditory feedback is provided to reinforce every correct response. In the detection task, targets are randomly presented within a stream of central fixation crosses. In the oddball task, targets are randomly presented within a stream of nontarget stimuli (low contrast Gabor patches). In the Go/NoGo task, Go stimuli (low frequency Gabor patch, 50%) and NoGo stimuli (high frequency Gabor patch, 50%) are randomly presented within a stream of central fixation crosses. Outcome measures for all tasks were omission errors (one minus correct responses to target stimuli), commission errors (anticipatory responses and responses to nontarget stimuli), and median reaction times to target stimuli.

### Statistical analysis

Questionnaire scores were standardized to published normative data. Raw scores on neuropsychological measures were transformed to study-specific *Z* scores. Behavioral data were checked for normality of distributions prior to parametric or nonparametric analysis. Full-scale IQ was included as a covariate in multivariate analyses.

### Neuroimaging acquisition and analysis

T1-weighted MP-RAGE, T2-weighted and FLAIR sequences were obtained on the Siemens 3 T Tim Trio system at the MRC Cognition and Brain Sciences Unit, Cambridge U.K. Qualitative reporting was carried out by a consultant neuroradiologist blind to genetic diagnosis.

Regional volumetric analysis was carried out via voxel-based morphometry (VBM). T1-weighted images underwent spatial preprocessing using the VBM8 Toolbox (Christian Gaser) in SPM8 (www.fil.ion.ucl.ac.uk/spm). Images were Dartel-realigned, normalized to the MNI template, segmented (modulated, affine and nonlinear), and smoothed at 8 mm Full Width at Half Maximum (FMHW) for gray matter (GM) and 12 mm FMHW for white matter (WM). Preprocessed images were visually inspected prior to case–control comparison, with co-variation for age and global GM or WM (voxel-wise tissue classification relative threshold 0.8, cluster threshold *k* = 50, statistical threshold *P* < 0.05 with family-wise error correction). Global tissue volumes (GM, WM, cerebrospinal fluid, intracranial volume) were extracted during preprocessing.

Corpus callosum midsagittal areas and subcortical structure volumes were traced in MRICron (Chris Rorden, www.mccauslandcenter.sc.edu/mricro/) using a graphics tablet. Landmarks for tracing the thalamus, caudate nucleus and putamen were defined by published methods.^27^

## Results

### Neurological histories

**Figure 1:**
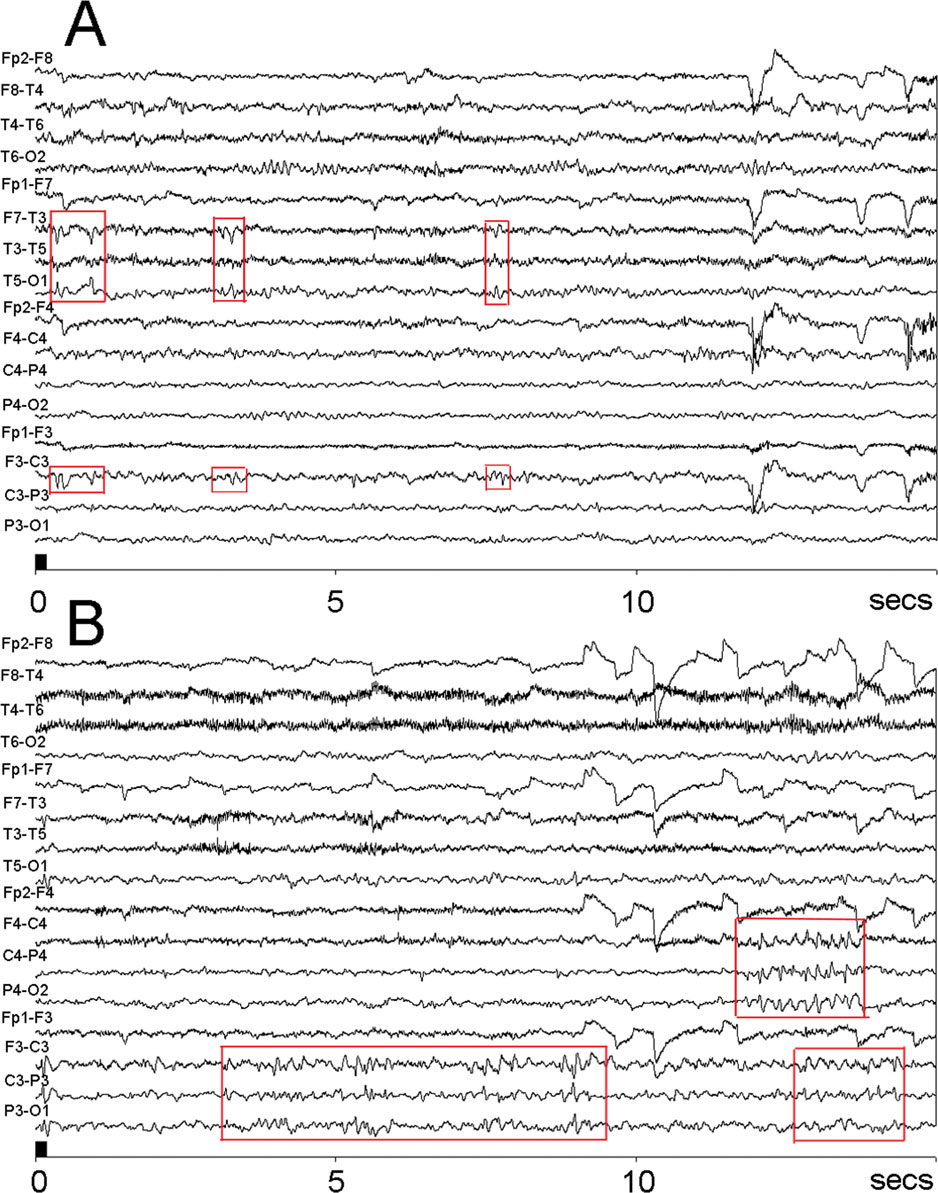
Clinical EEG recordings for two males with *ZDHHC9* mutations. (A) Recording at age 4 years (Case #8) to investigate suspected seizures, off anti-epileptic medication. Intermittent diphasic sharp waves, synchronous in left temporal and left central leads. (B) Recording at age 9 years (Case #7) to investigate suspected seizures, off anti-epileptic medication. Clusters of raised voltage diphasic sharp waves are maximal over the centro-parietal regions, more extensive on the left than right side.

All subjects were in good health at the time of assessment, with no reported deteriorations in neurological or cognitive function. A significantly higher proportion of *ZDHHC9* cases (7/9, 77.8%) than XLID controls (7/26, 26%) had received a diagnosis of epilepsy, according to documented local clinician evaluations of witnessed seizures and EEG (Pearson Chi-Square 7.2, *P* = 0.01). Seizure characteristics of *ZDHHC9* cases are described in Table S1, and EEG features are illustrated for two cases in Figure1. Seizures were brief (usually lasting several minutes) and self-terminating, occurring only or predominantly at night. Seizures were characterized by involuntary, sustained facial motor activity and speech arrest, with or without upper limb involvement, with secondary generalization in some cases. In one case, only generalized seizures were reported, however motor activity at onset of seizures had not been observed and the participant was not able to recollect preemptory symptoms, a limitation noted previously in idiopathic RE.^28^ Seizure onset was during midchildhood with remission during adolescence in all but one case (onset of focal seizures reported in early adulthood). One case had an atypically severe seizure history, presenting with an episode of status epilepticus after which he suffered from frequent focal seizures, infrequent brief generalized seizures, and night-time screaming episodes thought to be seizure-related. His seizures remitted by midadolescence. This individual manifested severe developmental delay with no regression, and severe intellectual disability with no speech.

### Behavioral questionnaires

8/9 *ZDHHC9* cases had mild or moderate ID and only 1/9 case had severe ID (Vineland Adaptive Behavior Composite range 20–71, mean 57, SD 15). 13/26 XLID controls had mild or moderate ID and 13/26 had severe or profound ID (Vineland ABC range 20–67, mean 40, SD 17). Hence, *ZDHHC9* cases had significantly milder global impairment than XLID controls on average (Mann–Whitney *U P* = 0.022). *ZDHHC9* cases showed prominent impairments in communication and motor abilities, not differing from XLID controls in these domains (Mann–Whitney *U* communication: *P* = 0.20; motor: *P* = 0.20) despite significantly stronger social and practical skills (Mann–Whitney *U*: socialization *P* = 0.003; daily living skills *P* = 0.019). Vineland communication subdomain scores indicated that the *ZDHHC9* case group had better receptive ability than expressive and written abilities, whilst XLID controls had better expressive ability than receptive and written abilities (main effect of group *F*_1,21_ = 0.88 *P* = 0.36, group × subdomain interaction *F*_2,42_ = 3.3 *P* = 0.047). There was no difference between groups in the profile of expressive impairments reported via Communication Checklists (main effect of group *F*_1,21_ = 0.43, *P* = 0.52, group × subdomain interaction *F*_2,42_ = 1.9 *P* = 0.16). There were no differences between groups in rates of behavioral problems.

### Cognitive testing

*ZDHHC9* cases and XLID controls who participated in cognitive testing were matched on verbal IQ and differed marginally on performance IQ and full scale IQ (FSIQ) (higher scores in *ZDHHC9* cases). After co-variation for FSIQ, no significant differences between *ZDHHC9* cases and XLID controls were observed on tests of auditory processing, receptive language functions, or sentence production (Table1).

**Table 1:** Standardized cognitive assessments

Domain (standardized battery)	Task	*ZDHHC9* cases, *n* = 8, mean (SD)	XLID controls, *n* = 9, mean (SD)	ANOVA, *F* (*P*)
General cognitive ability (WASI-II)	Full scale IQ (FSIQ),	65 (6)	57 (9)	5.4 (0.04)
	Perceptual reasoning index	67 (5)	59 (11)	3.7 (0.07)
	Verbal comprehension index	64 (6)	58 (11)	2.2 (0.16)
Auditory processing (SCAN-A)^1^	Filtered words	0.06 (0.67)	0.04 (1.61)	0.01 (0.9)^2^
	Competing words	0.1 (0.92)	−0.26 (1.38)	
Oromotor function (VMPAC)^1^	Focal oral control	−0.34 (1.1)	0.53 (0.61)	8.3 (0.016)^2^
	Sequencing	−0.13 (0.91)	0.21 (1.21)	
	Connected speech	−0.34 (0.92)	0.55 (0.96)	
	Quality of speech	−0.33 (1.1)	0.53 (0.53)	
Receptive language (CELF-IIIR)^1^	Concepts and directions	−0.13 (0.92)	0.20 (1.2)	3.9 (0.08)^2^
	Word CLASSES	−0.11 (0.83)	0.18 (1.3)	
Expressive language (CELF-IIIR)^1^	Formulating sentences	−0.02 (0.99)	0.03 (1.1)	0.87 (0.37)^2^
Rapid automatic naming (CELF-IIIR)^1^	Colors – time to complete	0.30 (0.96)	−0.15 (0.96)	0.32 (0.59)^2^
	Shapes – time to complete	0.11 (1.22)	−0.04 (0.70)	
	Colors + shapes – time to complete^3^	0.12 (1.29)	−0.14 (0.69)	

XLID, X-linked intellectual disability; ANOVA, analysis of variance; VMPAC, Verbal Motor Production Assessment for Children.

1 Full protocol completed by *n* = 8 *ZDHHC9* cases, *n* = 5 XLID controls; study-normalized Z scores.

2 Repeated Measures analysis for Domain, Covarying for FSIQ.

3 Not completed by *n* = 2 *ZDHHC9* subjects due to severe impairment on task.

The Rapid Automatic Naming test from CELF-IIIR highlighted verbal fluency problems amongst the *ZDHHC9* group not shared with XLID controls. Cases demonstrated a range of difficulties on this task including delayed initiation of responses, articulation problems, and difficulty inhibiting prior responses. For the color naming subtask, which all subjects could complete, the *ZDHHC9* case group took significantly longer than XLID controls to name all stimuli (Mann–Whitney *U P* = 0.045), with no difference in number of correctly named items.

The VMPAC assessment highlighted significant oromotor difficulties for the *ZDHHC9* group in comparison to XLID controls, affecting oral control, sequencing, voice characteristics and connected speech (main effect of group *F*_1,10_ = 8.3, *P* = 0.016, group × subscale interaction *F*_1,10_ = 0.17, *P* = 0.92). For oral control scores, the *ZDHHC9* group performed more poorly than XLID controls on both speech and nonspeech items (main effect of group *F*_1,10_ = 4.37, *P* = 0.06; post hoc analysis speech items *F*_1,10_ = 3.34, *P* = 0.09, nonspeech items *F*_1,10_ = 3.02, *P* = 0.11). For sequencing scores, there was no significant difference between groups (*F*_1,10_ = 2.46, *P* = 0.15) but post hoc analysis suggests that the *ZDHHC9* group is impaired in sequencing speech sounds (*F*_1,10_ = 4.62, *P* = 0.06) but not sequencing of nonspeech movements (*F*_1,10_ = 1.94, *P* = 0.19).

**Figure 2:**
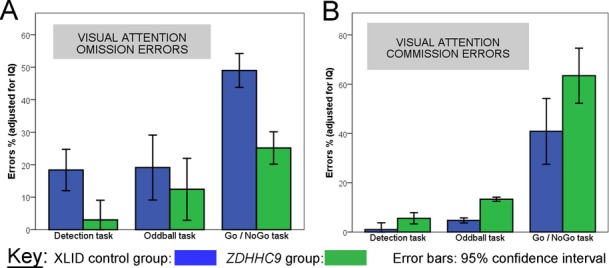
Attention performance in males with *ZDHHC9* mutations and ability-matched X-linked intellectual disability (XLID) controls. (A) Visual attention tasks, omission errors (one minus hit rate). (B) Visual attention tasks, commission errors (false alarms and anticipatory responses).

On visual attention tasks, the *ZDHHC9* group demonstrated lower omission and higher commission rates across all visual attention tasks, indicative of reduced inhibitory control (Fig.2). On the Go/NoGo task, the case group correctly responded to targets more often than controls, but were less successful at inhibiting responses to distractors within the Go/NoGo task main effect of group *F*_1,12_ = 0.76, *P* = 0.42, group × error-type *F*_1,12_ = 3.7, *P* = 0.08; post hoc analysis omission errors *F*_1,10_ = 1.96, *P* = 0.19, commission errors *F*_1,10_ = 3.55, *P* = 0.08). There was no differences between groups in speed of responding (*F*_1,12_ = 0.26, *P* = 0.63, group × task *F*_1,12_ = 0.8, *P* = 0.93), indicating that generalized motor differences do not underlie case-control differences in attentional performance.

### Neuroimaging

**Figure 3:**
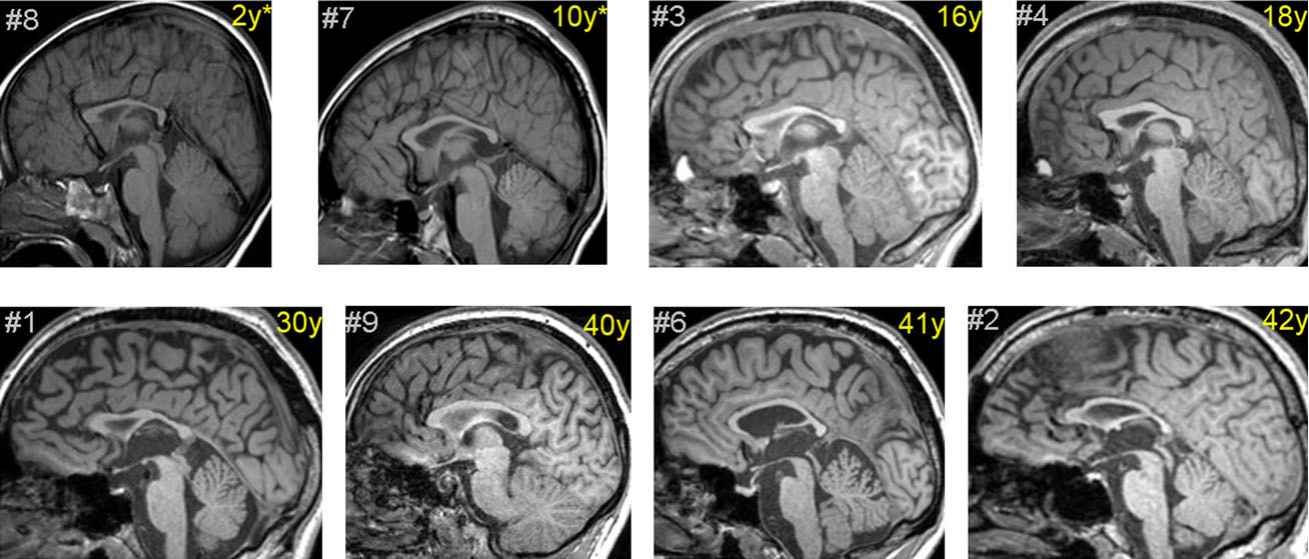
Magnetic resonance imaging for males with *ZDHHC9* mutations. T1-weighted MRI (midsagittal slice) for eight males with *ZDHHC9* mutations. Scans are ordered by chronological age at the time of MRI image acquisition. *Clinically acquired scans. #Case number in Table S1.

Qualitative rating blind to diagnosis identified global or segmental hypoplasia of the corpus callosum in seven *ZDHHC9* mutation cases, global cerebral volume loss in five cases, ventricular enlargement in six cases and cerebellar vermis volume reduction in one case (Fig.3). No focal parenchymal anomalies were noted. None of these features was present in any healthy age-matched comparison subject. Hypoplasia of the corpus callosum was a stable phenomenon for one case with clinical neuroimaging available at multiple ages (6, 10 and 13 years) and was present prior to the onset of seizures in two cases scanned during early childhood.

**Figure 4:**
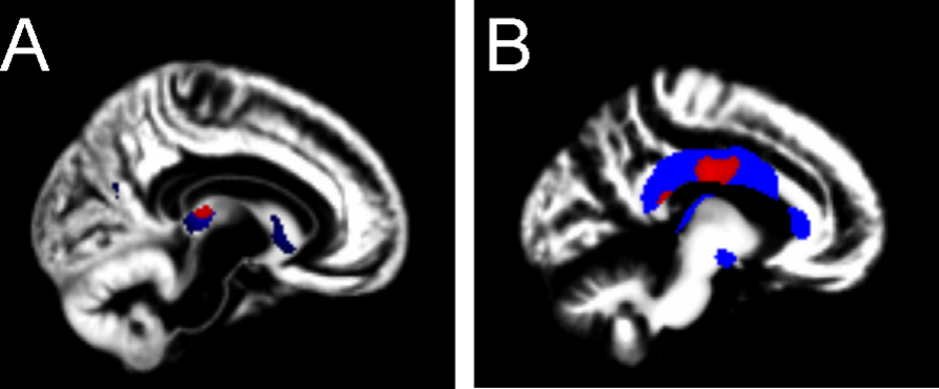
Voxel-based morphometry. Comparison of regional volumetric differences between males with *ZDHHC9* mutations (*n* = 7) and age-matched male controls (*n* = 7). Background = mean normalized segmented image for all study participants. (A) Regional gray matter reductions; red = *ZDHHC9 *<* *controls (*P* < 0.05_FWE_), blue = *ZDHHC9 *<* *controls (*P* < 0.001_uncorrected_). (B) Regional white matter reductions; red = *ZDHHC9 *<* *controls (*P* < 0.05_FWE_), blue = *ZDHHC9 *<* *controls (*P* < 0.001_uncorrected_).

Quantitative comparison indicated no significant differences in intracerebral volume (*P* = 1.0) or GM volume (Mann–Whitney *U P* = 0.62) but a trend toward reduced WM volume (*P* = 0.13) and increased cerebrospinal fluid volume (*P* = 0.07) in the case group. VBM analysis (Fig.4) identified significant bilateral GM reductions in the thalamus. At uncorrected statistical threshold, GM reductions in the left caudate were also seen. No areas of cortical GM difference survived correction for multiple comparisons. WM reductions were maximal in the body of the corpus callosum, extending into the temporal lobes bilaterally. There were no significant regions of relative WM increase for *ZDHHC9* cases.

**Figure 5:**
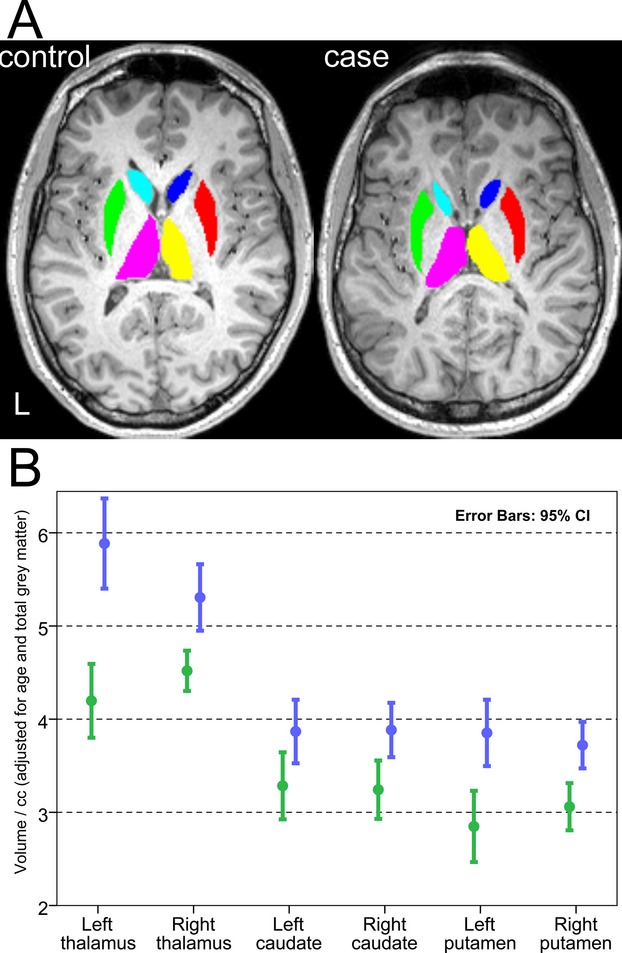
Region-of-interest measurements. (A) Example tracings of subcortical structures at an equivalent axial level on T1-weighted imaging for a single *ZDHHC9* mutation case and their age-matched control. Structures were traced from inferior to superior slices according to predefined landmarks to capture entire volumes. Pale blue = left caudate, dark blue = right caudate, green = left putamen, red = right putamen, pink = left thalamus, yellow = right thalamus. (B) Quantification of subcortical volumes for males with *ZDHHC9* mutations and healthy age-matched controls, adjusted for age and total gray matter volume.

Comparison of region-of-interest measurements (Fig.5) was consistent with automated VBM analysis. On average, corpus callosum cross-sectional area was reduced by 52% in *ZDHHC9* cases versus controls, thalamic volume by 28.8% on left and 14.9% on right, caudate volume by 15.2% on left and 16.5% on right, and putamen volume by 26.2% on left and 26.0% on right. Cross-sectional area of the corpus callosum was significantly reduced in absolute values (Mann–Whitney *U P* = 0.001), relative to intracranial volume (*P* = 0.001), and relative to global WM (*P* = 0.001), with no outliers and a narrow distribution of values within the *ZDHHC9* group. Repeated measures ANOVA was used to compare bilateral thalamic, caudate and putamen volumes, covarying for age and total GM. This confirmed a highly significant difference between groups (*F*_1,11_ = 29.7, *P* < 0.001), with no interaction between group and structure (*F*_2,22_ = 2.28, *P* = 0.13), or group and hemisphere (*F*_1,11_ = 2.6, *P* = 0.13).

## Discussion

This study demonstrates that genetic diagnosis in neurodevelopmental disorders, including intellectual disability and common childhood seizure disorders, will have enhanced prognostic value and therapeutic relevance when coupled to multimethod phenotyping. Specifically, we have observed association between a recurrent X-linked cause of ID, focal seizures with similarity to the RE spectrum, and long-term linguistic and nonlinguistic deficits. Unlike the idiopathic (mixed etiology) RE population where cognitive outcomes are variable and neuroanatomical differences are either subtle or nonapparent, *ZDHHC9* mutations are associated with relatively homogeneous cognitive and neuroanatomical abnormalities, highlighting a novel developmental pathway for future exploration.

Ascertainment of *ZDHHC9* cases was via genetic screening of an XLID cohort*,* a strategy with inherent benefits and limitations. Retrospective documentation of epilepsy phenotypes was not comprehensive and EEG recordings from the time of active epilepsy could not be traced for all cases. Cognitive and behavioral comparisons are less informative across a wide age range. Pooling of phenotype data from individuals with and without current seizures, and from individuals with and without current antiepileptic medication may introduce confounding factors which can only be addressed in a larger study. Despite these limitations and potential confounding factors, results were consistent across the study group. We find a higher degree of consistency in neurological and cognitive characteristics than in physical characteristics associated with *ZDHHC9* mutations – only three out of five reported families include individuals with skeletal features reminiscent of marfanoid habitus, whereas all families include individuals with epilepsy, oromotor impairments, intellectual disability, and abnormal corpus callosum; neurocognitive features more reliably segregate with mutations within families than does dysmorphology.^3^,^29^ In general, systematic phenotyping of neurological and cognitive functions may assist interpretation of genomic test results more reliably than physical dysmorphology in neurodevelopmental disorders.

Epilepsy prevalence amongst males with *ZDHHC9* mutations was at least three times that expected for ID overall and seven times that expected for mild ID,^30^ significantly exceeding prevalence amongst comparison subjects with XLID. The reported ictal phenomena and natural history amongst *ZDHHC9* cases suggest susceptibility to focal seizures sharing features with RE, with a range of seizure severity in keeping with observations across the idiopathic RE spectrum including previous family studies.^7^,^31^ Childhood seizures associated with centrotemporal spikes on EEG were recently reported for one further male with a *ZDHHC9* truncating mutation, providing further independent evidence for this association.^29^ With one exception, age of onset and offset of seizures amongst *ZDHHC9* mutations cases was in keeping with the typical maturational profile of RE. The identification of a single *ZDHHC9* mutation case with focal seizures during adulthood could reflect different pathophysiology of epilepsy in the *ZDHHC9* group versus idiopathic RE, or underreporting of late seizures in individuals susceptible to RE, or an unrelated pathology in this case. Where EEG data were available for review, neurophysiological features were in keeping with an RE spectrum disorder, with variable interictal phenomena similar to observations in the idiopathic population.^7^ For two adult cases where neurophysiological data were no longer available, local clinician evaluation of clinical history and EEG at the time of active seizures had led to diagnosis and management of an RE spectrum disorder. In future prospective studies, acquisition of sleep EEG would be desirable to demonstrate sleep activation of epileptiform discharges, reduction in mu rhythms and muscle artifact, and modeling of dipole sources.

It is highly unlikely that one-to-one relationships will ever be established between genetic etiologies, electrophysiological abnormalities and clinical epilepsy syndromes, as illustrated by recent identification of *GRIN2A* mutations*, DEPCD5* mutations and 16p11.2 duplications across a number of focal epilepsies and epilepsy aphasia syndromes.^32^–^35^ We suggest that *ZDHHC9* mutation is associated with susceptibility to focal seizures, but at this point cannot be more precise about the range of electroclinical phenotypes that may be associated with mutations in this gene. This issue can only be addressed via empirical investigation to establish the rates of *ZDHHC9* mutations amongst individuals ascertained for different epilepsy syndromes.

Having detected the unexpectedly high frequency of focal epilepsy amongst males with *ZDHHC9* mutations, we posed the hypothesis that specific cognitive deficits showing similarity to idiopathic RE might differentiate *ZDHHC9* from other causes of XLID. The hypothesis was supported: expressive communication, particularly oromotor control and fluent speech production, was a prominent area of difficulty for *ZDHHC9* cases, consistent with previous observations of verbal dyspraxia and speech processing disorders in familial and sporadic RE.^8^,^10^,^36^,^37^ The standardized VMPAC assessment, rated blind to genetic diagnosis, highlighted deficits in speech sound production and nonspeech movements such as control of tongue movements and orolingual sequences, consistent with independent clinical reports of oral movement abnormalities in two *ZDHHC9* mutation cases.^29^ We also observed differences between the *ZDHHC9* and XLID comparison groups on measures of nonlinguistic visual attention, with the *ZDHHC9* group showing higher rates of false alarm errors, suggestive of impairment to inhibitory control mechanisms. A very similar result – increased commission errors but not omission errors on a Go/NoGo task – has been reported for children with RE^14^ but not at-risk siblings,^8^ suggesting a relationship between rolandic seizure activity and the development of attentional control networks. Cognitive impairments amongst the *ZDHHC9* group were present irrespective of overt seizure history, were present prior to the onset of overt seizures, and were persistent many years after seizure remission, as recently documented for idiopathic RE.^12^

An important caveat is that cognitive deficits in males with *ZDHHC9* mutations were more uniform than previously observed in RE and occur on a background of mild to moderate ID. There are several possible explanations for the higher degree of comorbidity between seizure susceptibility and cognitive impairments amongst males with *ZDHHC9* mutations as compared to idiopathic RE. Firstly, impairments may reflect an etiology-specific developmental pathway, overlapping with some but not all individuals with RE. Around one-third of idiopathic RE cases have a speech processing disorder,^10^ and this subset of the RE population may share pathophysiology with *ZDHHC9* at the levels of molecular, cellular or neural network mechanisms. Alternatively, observed differences between the genetic and idiopathic populations may reflect methodological differences between studies. Ascertainment for XLID in the current study may have introduced bias toward cognitive comorbidities, and exclusion of individuals with ID from studies of BECTS will have resulted in the opposite bias. Nevertheless, the choice of an ability-matched XLID comparison group in the current study, rather than reliance on general population standardization, has enabled specific deficits to be highlighted.

As a first step toward identifying mechanisms underlying the *ZDHHC9-*associated neurocognitive phenotype we obtained structural MRI data for all but the most severely intellectually impaired case and identified consistent neuroanatomical abnormalities. Quantitative analysis revealed focal GM abnormalities within subcortical structures, maximal in the thalamus. Thalamic abnormalities have not previously been associated with RE, but have been detected in other pediatric epilepsy syndromes.^38^,^39^ Subcortical abnormalities are also the hallmark of inherited speech motor disorder caused by *FOXP2* mutations.^40^ In addition, we and others have observed hypoplasia of the corpus callosum (generalized or segmental) to be a very consistent finding in *ZDHHC9* cases, at every age from early childhood to midadulthood.^29^ Hypoplasia of the corpus callosum has only rarely been reported in idiopathic RE^41^ however recent application of diffusion-weighted imaging has highlighted inter-hemispheric changes that correlate with duration of RE and with cognitive impairments,^42^ suggesting possible convergence between severe, homogeneous structural differences in *ZDHHC9* and more subtle differences in a proportion of the idiopathic RE population. In the adult human brain, ZDHHC9 expression is restricted to the globus pallidus, ventral thalamus and WM, with low expression throughout the cerebral cortex,^43^ convergent with observed structural abnormalities in *ZDHHC9* mutation cases. Developmental transcriptome data indicate regionally-specific and temporally-specific regulation of *ZDHHC9* expression, with thalamic expression being maximal during antenatal life, and striatal expression increasing to maximal levels during adulthood (http://brainspan.org/).

One explanation for the differences between neuroimaging findings in *ZDHHC9* cases and idiopathic RE could be the low IQ of the case group-features could be nonspecific observations associated with ID, not related to etiology or specific cognitive phenotype. In keeping with this proposal, the cross-sectional area of the corpus callosum correlated with IQ in a follow-up study of preterm infants^44^ and a similar relationship has been observed in the general population.^45^ However, in two genetic syndromes with similar IQ reductions to the *ZDHHC9* group*,* we observed age-appropriate callosal area or relative callosal enlargement, indicating that callosal hypoplasia is not a universal abnormality in ID.^46^,^47^ Thinning of the corpus callosum and especially of posterior connections between temporal cortices appears to be a common observation amongst neurodevelopmental disorders with prominent expressive language impairment, whether due to genetic cause, for example *ARID1B*^48^, or nongenetic cause, for example prematurity.^49^ Hence we speculate that both callosal and subcortical abnormalities in the *ZDHHC9* case group reflect disruptions to developmental connectivity convergent with other language-impaired populations.

The consequences of ZDHHC9 loss-of-function for neuronal excitability and maturation of subcortical–cortical systems have yet to be established. Regulators of neuronal excitability known to be palmitoylated include the voltage-gated sodium channel^50^ and BK channels.^51^ In addition, palmitoylation regulates the synaptic targeting of PSD-95, a key component of the postsynaptic scaffolding apparatus which regulates NMDA receptor availability.^52^,^53^ *ZDHHC9* may be an informative single gene model for investigation of activity-dependent network maturation relevant to epileptogenesis, speech-motor function and higher order cognitive development.

For individuals diagnosed with *ZDHHC9* mutations, results of this study have prognostic significance since epilepsy can be anticipated and support can be targeted to promote expressive communication skills. For the RE population, genetic testing for *ZDHHC9* and other monogenic causes may yield further diagnoses with potential to explain variability in cognitive outcomes. In time, prospective developmental comparisons between different monogenic causes of RE spectrum disorders may be informative of distinct neurodevelopmental trajectories, reflecting molecular pathways which may be amenable to therapeutic modification.

## Supporting Information

**Table S1.** Epilepsy histories, EEG features and neurological examinations in males with *ZDHHC9* mutations.
