# Supplementary material for: Epilepsy, cognitive deficits and neuroanatomy in males with ZDHHC9 mutations
Source: Ann Clin Transl Neurol. 2015 Apr 9;2(5):559–69. doi: 10.1002/acn3.196 (PMC4435709; doi:10.1002/acn3.196)
Supplement: Supplementary file 1 — Table S1. Epilepsy histories, EEG features and neurological examinations in males with ZDHHC9 mutations. [file acn30002-0559-sd1.docx]

**Supplemental Table 1. Epilepsy histories, EEG features and neurological examinations in males with *ZDHHC9* mutations**

| ***Case*** | **#1** | **#2** | **#3** | **#4** | **#5** | **#6** | **#7** | **#8** | **#9** |
| --- | --- | --- | --- | --- | --- | --- | --- | --- | --- |
| ***Mutation*** | c.442C→T | c.442C→T | c.172_175dup | c.172_175dup | c.172_175dup | c.448C→T | c.448C→T | c.448C→T | c.448C→T |
| ***Age at assessment*** | 32 yrs | 42 yrs | 16 yrs | 18 yrs | 37 yrs | 41 yrs | 13 yrs | 9 yrs | 38 yrs |
| ***Age at seizure onset*** | No seizures | 8 yrs | 6 yrs | No seizures | 4 yrs | 30yrs | 9 yrs | 4 yrs | 9yrs |
| ***Age at last seizure*** | n/a | 14 yrs | 14 yrs | n/a | <20 yrs | Ongoing | Ongoing | Ongoing | 13yrs |
| ***Seizure frequency*** | n/a | Fewer than 10 total | Sporadic clusters | n/a | Initially frequent then 2-3 per year | 3-4 per month | 3-4 per month | Sporadic clusters | Fewer than10 total |
| ***Seizure timing*** | n/a | Nocturnal | Nocturnal | n/a | Diurnal | Mainly nocturnal | Nocturnal | Daytime | Daytime |
| ***Seizure types*** | n/a | Focal orofacial with brief generalisation | Focal orofacial with brief generalisation | n/a | Focal upper limbs and generalised. One episode status epilepticus. | Focal orofacial and upper limb | Focal orofacial and right upper limb | Speech arrest and dysarthria | Brief generalised |

| ***Case*** | **#1** | **#2** | **#3** | **#4** | **#5** | **#6** | **#7** | **#8** | **#9** |
| --- | --- | --- | --- | --- | --- | --- | --- | --- | --- |
| ***Clinical EEG*** | Not done | Bilateral sharp wave discharges | Bilateral centrotemporal sharp waves | Not done | No longer available | Not done | Bilateral centroparietal sharp waves | Bilateral centrotemporal sharp waves | No longer available |
| ***Current medication (past)*** | Nil | Nil (Carbamazepine) | Carbamazepine (Melatonin) | Nil | Carbamazepine (sodium valproate) | Phenytoin | Carbamazepine and lamotrigine | Nil (lamotrigine) | Nil |
| ***Adaptive level*** | Mild ID | Moderate ID | Mild ID | Mild ID | Severe ID | Mild ID | Mild ID | Mild ID | Moderate ID |
| ***Neurology exam deficits*** | Poor balance | Poor control of tongue movements. Inaccurate motor sequencing. Poor balance. | Limited facial movements. Inaccurate motor sequencing. Poor balance. | Poor balance | Limited facial movements. Not ambulant. | Hypemetropia. Poor balance. | Hypermetropia. Limited facial / tongue movements. Poor balance. | Hypermetropia. Poor balance. | Hypermetropia. Inaccurate motor sequencing. Poor balance. |
